# MRI-guided focal laser ablation for prostate cancer followed by radical prostatectomy: correlation of treatment effects with imaging

**DOI:** 10.1007/s00345-016-1924-1

**Published:** 2016-08-19

**Authors:** Joyce G. R. Bomers, Erik B. Cornel, Jurgen J. Fütterer, Sjoerd F. M. Jenniskens, H. Ewout Schaafsma, Jelle O. Barentsz, J. P. Michiel Sedelaar, Christina A. Hulsbergen-van de Kaa, J. Alfred Witjes

**Affiliations:** 1 Department of Radiology, Radboud University Medical Center, P.O. Box 9101, 6500 HB Nijmegen, The Netherlands; 2 Department of Urology, Ziekenhuisgroep Twente, P.O. Box 546, 7550AM Hengelo, The Netherlands; 3 MIRA Institute for Biomedical Technology and Technical Medicine, University of Twente, Building Zuidhorst, P.O. Box 217, 7500 AE Enschede, The Netherlands; 4 Department of Pathology, Radboud University Medical Center, P.O. Box 9101, 6500 HB Nijmegen, The Netherlands; 5 Department of Urology, Radboud University Medical Center, P.O. Box 9101, 6500 HB Nijmegen, The Netherlands

**Keywords:** Prostate cancer, MRI, Focal therapy, Laser ablation

## Abstract

**Purpose:**

To correlate treatment effects of MRI-guided focal laser ablation in patients with prostate cancer with imaging using prostatectomy as standard of reference.

**Methods:**

This phase I study was approved by the Institutional Review Board. Three weeks prior to prostatectomy, five patients with histopathologically proven, low/intermediate grade prostate cancer underwent transrectal MRI-guided focal laser ablation. Per patient, only one ablation was performed to investigate the effect of ablation on the tissue rather than the effectiveness of ablation. Ablation was continuously monitored with real-time MR temperature mapping, and damage-estimation maps were computed. A post-ablation high-resolution T1-weighted contrast-enhanced sequence was acquired. Ablation volumes were contoured and measured on histopathology specimens (with a shrinkage factor of 1.15), T1-weighted contrast-enhanced images, and damage-estimation maps, and were compared.

**Results:**

A significant volume correlation was seen between the ablation zone on T1-weighted contrast-enhanced images and the whole-mount histopathology section (*r* = 0.94, *p* = 0.018). The damage-estimation maps and histopathology specimen showed a correlation of *r* = 0.33 (*p* = 0.583). On histopathology, the homogeneous necrotic area was surrounded by a reactive transition zone (1–5 mm) zone, showing neovascularisation, and an increased mitotic index, indicating increased tumor activity.

**Conclusions:**

The actual ablation zone was better indicated by T1-weighted contrast-enhanced than by damage-estimation maps. Histopathology results highlight the importance of complete tumor ablation with a safety margin.

## Introduction

With an estimated amount of 233,000 newly diagnosed patients and 29,000 deaths in the United States in 2014, prostate cancer (PCa) has a significant impact on society [1]. The treatment choice for low-to-intermediate grade PCa patients ranges between active surveillance and whole-gland therapies, i.e., radical prostatectomy or radiotherapy. However, these treatments come with morbidity and influence quality-of-life (QoL) [2, 3]. For instance, 2 years after treatment, 3.2–9.6 % of these patients experience urinary leakage and 56–78.8 % suffers from erectile dysfunction [2, 3].

Focal therapy is an emerging alternative treatment option for low-to-intermediate grade PCa [4]. It is a strategy, by which the overtreatment burden of the current prostate cancer pathway could be reduced, and QoL is preserved. The challenge is to treat the tumor entirely, sparing normal prostate tissue, especially near the neurovascular bundles and the urethral sphincter, and to minimize potential morbidity. Currently, focal therapy should be performed under strict surveillance, because of its experimental nature.

Various minimal invasive techniques, such as cryoablation, high-intensity focused ultrasound (HIFU), irreversible electroporation, and focal laser ablation (FLA), have been used to perform focal therapy in PCa patients [5–11]. All these techniques have their own characteristics and are still evolving. At this moment, there is no consensus which technique is optimal for focal therapy.

Of the available minimal invasive focal therapy techniques, MRI-guided FLA is considered a promising option, since it is fast, creates a sharply defined ablation zone, is minimally invasive for the patient, and can be performed under local anesthesia in an outpatient setting. During this treatment, a laser fiber is inserted into the tumor and the tissue is irreversibly damaged and destroyed when its temperature increases >60 °C. The total ablation process takes only a few minutes. Furthermore, repeat treatments, as well as secondary radical treatment, are still possible. MRI guidance appears essential in FLA and might prove to be the only imaging modality for correct targeting of the index lesion, facilitating accurate fiber placement, real-time monitoring of the ablation with the help of temperature mapping, and verification of complete tumor ablation [12].

To date, only a few studies reported on MRI-guided FLA in PCa patients [5, 13–17]. Moreover, the latter include feasibility studies using small sample sizes and follow-up ≤1 year. One study validated the ablation volume after ultrasound-guided FLA, but used a laser system with a different wavelength [12]. Validation of MRI-guided FLA is required, before it can gain acceptance as treatment option for patients with low-to-intermediate grade PCa. Insight into the actual treatment zone can be obtained by comparing per-procedural MRI-temperature maps and histopathology correlation.

Therefore, the goal of our study was to correlate treatment effects of MRI-guided FLA, rather than treatment success. Before radical prostatectomy, patients underwent MRI-guided FLA as extra treatment. One single ablation per tumor was performed to investigate the effect of the ablation on the tissue, rather than the effectiveness of the treatment. Damage-estimation maps derived of MRI-temperature maps, post-ablation T1-weighted contrast-enhanced (T1wCE) MR images, and histopathology specimens were used to correlate the expected and actual size of the ablated region.

## Materials and methods

### Patients

**Fig. 1:**
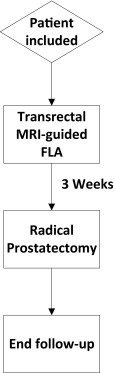
Study flow diagram

The study was approved by the Institutional Review Board (IRB) and all patients provided written informed consent. Between May 2012 and November 2013, five patients were included. A study flow diagram is shown in Fig. 1. Inclusion criteria were men with newly diagnosed and histopathologically proven PCa with a prostate-specific antigen (PSA) level of ≤20 ng/mL, Gleason score ≤7, stage ≤T2b, no previous treatment for PCa, scheduled for radical prostatectomy, and a tumor lesion visible on the anatomical T2-weighted (T2w) and/or diffusion-weighted images (DWI) of the diagnostic multi-parametric MRI and located more than 2 cm of the neurovascular bundle. Patients with contra-indications to MRI or MRI-guided FLA, nodal or metastatic disease, or an estimated Glomerular Filtration Ratio <40 mL/min/1.73 m^2^ were excluded.

### MRI-guided focal laser ablation

Patients were prescribed antibiotic prophylaxis (ciprofloxacin) for three days, starting on the day before the FLA procedure. All procedures were performed on a 3 Tesla MRI scanner (TrioTIM, Siemens, Erlangen, Germany) and a 980 nm diode laser system (Visualase, Houston, Texas, USA). Patients were positioned head first in prone position on the scanner table. A pelvic phased-array coil was placed over the patient’s pelvis.

A gadolinium-filled needle guide was inserted in the rectum and attached to an MRI-compatible device (DynaTrim, Invivo, Schwerin, Germany), originally developed for MRI-guided biopsies. The procedure was performed under local anaesthetic block administered by bilateral injections of lidocaine between the base of the prostate and the seminal vesicles [18]. Anatomical T2w-images and DWI were acquired to re-identify the MRI visible lesion. All image parameters are shown in Table 1. TrueFisp images were acquired in two directions, to direct the needle guide to the lesion. After correct alignment, a 14-gauge catheter with a titanium trocar was inserted through the needle guide. Once the catheter was positioned inside the tumor, the titanium trocar was replaced by the laser fiber. The tip of the laser fiber was unsheathed from the catheter to enable immediate contact with the tumor tissue. Proper laser fiber placement was verified in two ways: first by acquiring a TrueFisp sequence and second by applying laser energy on a reduced power level. This was insufficient to cause thermal injury; however, this was visible on the MRI thermometry images.

**Table 1:** MRI parameters

Proto-col	Plane	TA (min:s)	TR (ms)	TE (ms)	FA (°)	No of slices	Slice thickness (mm)	Voxel size (mm × mm)	FOV (mm × mm)	Matrix size	Ave-rages	Band-width (Hz/Px)	b-values (s/mm^2^)	Temporal resolution (s)
*Laser ablation protocol (Siemens MAGNETOM TrioTIM)*														
T2WI TSE	Axial	3:35	4570	101	120	19	3.0	1.1 × 0.8	256 × 256	320 × 224	2	98	n.a.	n.a.
DWI EPI	Axial	3:08	2500	64	n.a.	20	4.0	2.0 × 2.0	256 × 212	128 × 128	8	1220	50/500/800	n.a.
True-fisp SSFP	Axial Sag	8.9 7.5	4.48 4.48	2.24 2.24	70 70	5 5	3.0 3.0	1.1 × 1.1 1.1 × 1.1	280 × 280 280 × 280	256 × 256 256 × 256	1 1	558 558	n.a. n.a.	n.a. n.a.
T2WI Haste	Axial	0:18	1800	94	120	10	3.0	1.7 × 1.3	340 × 276	256 × 205	1	781	n.a.	n.a.
T-MAP		n.a.	65	20	30	n.a.	5.0	2.0 × 1.0	260 × 260	256 × 128	1	260	n.a.	4.95
T1WI TSE	Axial	3:02	700	11	150	19	3.0	1.0 × 0.8	200 × 200	256 × 192	1	178	n.a.	n.a.
*Detection protocol 3T (Siemens MAGNETOM Skyra)*														
T2WI TSE	Sag Axial Cor	2:53 4:15 1:57	5590 5660 4320	101 104 101	160 160 160	19 19 15	3.0 3.0 3.0	0.6 × 0.6 0.5 × 0.5 0.6 × 0.6	180 × 180 192 × 192 192 × 192	320 × 320 384 × 384 320 × 320	3 4 2	200 200 200	n.a. n.a. n.a.	n.a. n.a. n.a.
DWI EPI	Axial	4:08	3200	63	n.a.	19	3.0	2.0 × 2.0	256 × 240	128 × 128	8	1502	50/500/ 800/ calc 1400	n.a.
DCE 3D PD GE	Axial	2:13	800	1.53	14	16	3.0	1.5 × 1.5	192 × 192	128 × 128	1	570	n.a.	n.a
	Axial	2:34	36	1.41	14	16	3.0	1.5 × 1.5	192 × 192	128 × 128	1	750	n.a	3.5

*TA* acquisition time, *TR* repetition time, *TE* echo time, *FA* flip angle, *FOV* field of view, *T2WI* T2-weighted imaging, *TSE* turbo-spin echo, *DWI* diffusion-weighted imaging, *EPI* echo planar imaging, *SSFP* steady-state free precession, *Sag* sagittal, *HASTE* half-fourier acquisition single-shot turbo-spin echo, *T-MAP* temperature map, *T1W1* T1-weighted imaging, *DCE* dynamic contrast-enhanced imaging, *PD* proton density, *GE* gradient echo

**Fig. 2:**
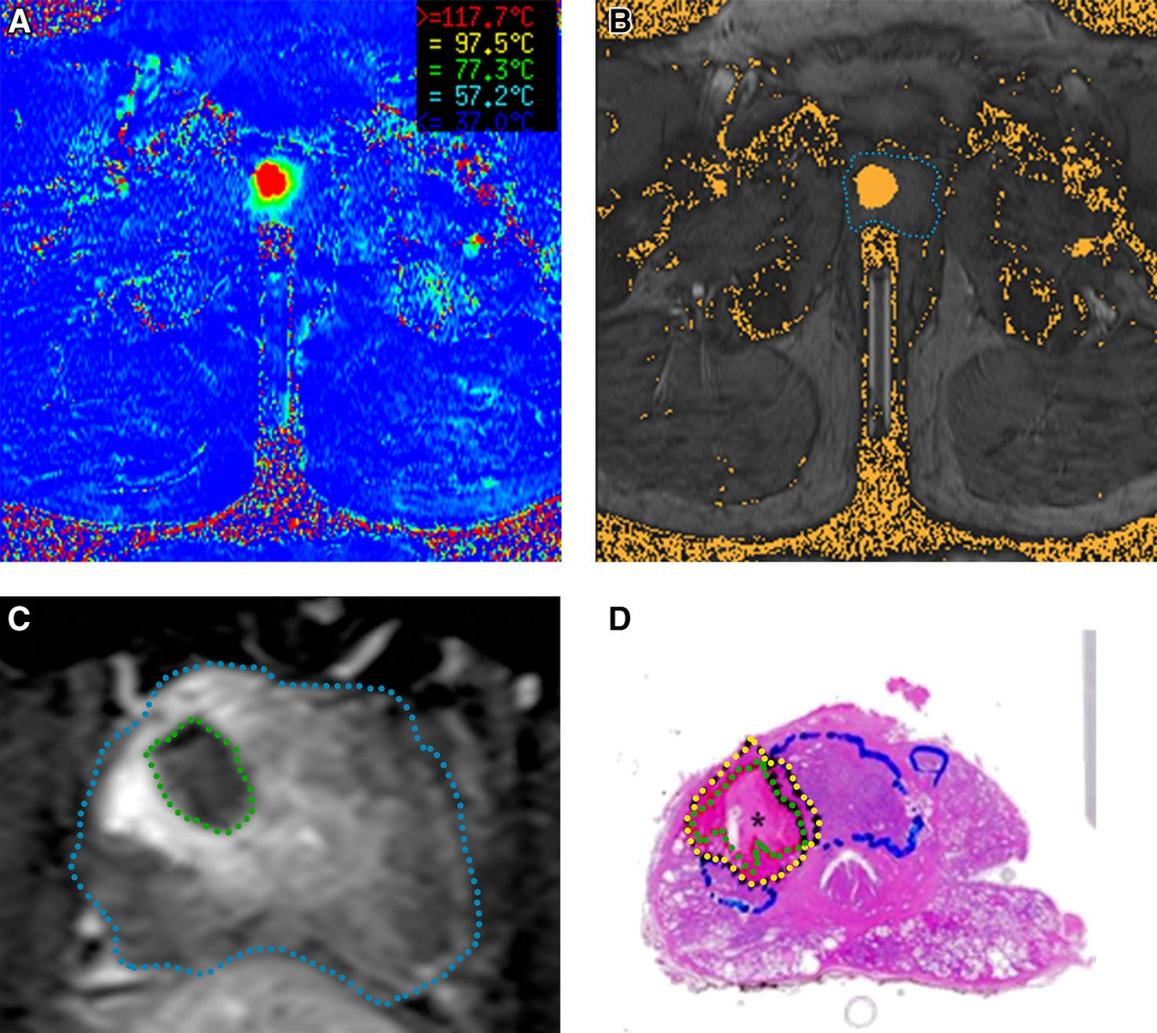
Images of a 67-year-old male with a Gleason score 3 + 4 = 7 in his right transition zone. **a** Real-time MRI-temperature map acquired during FLA. **b** Anatomical MRI of the prostate (*delineated* in *blue*) with the Arrhenius-based damage-estimation zone as orange overlay. **c** Post-ablation axial T1-weighted contrast-enhanced image. The prostate is *delineated* in *blue* and the non-enhancing necrotic tissue in *green*. **d** Whole-mount H&E stain of the prostate. The necrotic tissue is *delineated* in *green*, the perinecrotic tissue in *yellow,* and vital tumor in *blue*

The FLA procedure was continuously monitored with MRI thermometry images, acquired every 5 s in a single plane through the laser fiber. Relative temperatures were calculated based on the proton resonance frequency (PRF) method [19], and the temperature maps (Fig. 2a) were shown on the integrated standalone Visualase workstation connected to the MRI scanner. If the temperature at a certain point increased too much, this was immediately visible. Next to the temperature maps, a damage-estimation zone with an estimation of the final ablation area was shown as an overlay on the anatomic MRI (Fig. 2b). This zone was computed using the Arrhenius model for thermal tissue ablation [20].

Tumors were only partially ablated, because per patient only one ablation was performed to investigate the effect of the ablation on the tissue rather than the effectiveness of the treatment.

Immediately after ablation, an axial T1-weighted contrast-enhanced (T1wCE) sequence was performed (Fig. 2c) to assess the size of the ablation. Hereafter, the laser fiber and the needle guide were removed. Patients were discharged when voiding was uncomplicated.

Procedure time was determined as from the moment the first MRI sequence was started until the last MRI sequence was finished, including T2w and DWI, to re-identify the tumor and T1wCE images to assess ablation zone. Three weeks after FLA, patients underwent open radical prostatectomy, performed by one of two oncologic urologists with 25 and 9 years experience.

### Histopathology work-up

The resected prostate specimens were fixed overnight in 10 % neutral-buffered formaldehyde and coated with ink. Transverse whole-mount step sections were created at 4 mm intervals in a plane parallel to the axial plane used to perform the T1-weighted sequence. Apical and basal slices were additionally sliced in the sagittal plane and all sections were embedded in paraffin. Tissue sections of 5 μm were stained with hematoxylin-eosin (H&E) (Fig. 2d). The presence and extent of necrotic tissue, the surrounding perinecrotic zone with reactive changes, and vital tumor were annotated on the glass cover with the tissue section. In addition, immunostaining for CK8/18, CD31, and Ki67 was performed to demonstrate the presence of epithelial damage, vascular damage, and mitotic activity. The mitotic index was determined as the mean count of Ki67-positive nuclei in five microscopic fields of 1 mm^2^ surface area (20× objective Zeiss Axioskop). The fields for counting were taken in the first 1 mm zone of vital tumor (or pre-existing prostatic ducts) directly adjacent to the necrotic area; at the opposite tumor invasion front; and in the center of the tumor more than 2 mm distant from the perinecrotic or invasion front areas. For tumor tissue, areas were chosen with the same Gleason score and duct density. All measurements were independently performed by two genitourinary pathologists with 22 and 27 years experience.

### Data analyses

By assuming that the final form of the Arrhenius-based damage-estimation zone would be ellipsoid, the length and width of the final zone were measured and the ablation volume was calculated. Two experienced prostate MRI radiologists with 11 and 3 years experience blinded for the final pathology results contoured the ablation zone per slice in the post-ablation T1wCE images. The ablation area per slice was calculated by the PACS software and multiplied with the slice thickness to determine the total ablation volume. Volumes of the necrotic zone annotated on the histopathology slices were measured using an in-house developed software program. A factor of 1.15 was used to correct for tissue shrinkage [21].

Per patient, the volume of the ablated tissue in the prostate specimen was compared with the volume of the Arrhenius-based ablation zone and the volume of the ablation zone in the T1wCE images by calculating the ratios between the volumes. Statistical comparisons were made by calculating the Pearson correlation coefficient r and corresponding two-tailed *p* values with SPSS Statistics version 20. *P* values <0.05 were considered statistically significant.

## Results

### Patients

MRI-guided FLA was safe and feasible to perform and no immediate complications were encountered. All patients were discharged 1 h after treatment.

Two days after the MRI-guided FLA procedure, one patient was hospitalized for 1 week with urosepsis. He was treated with intravenous antibiotics and underwent radical prostatectomy 3 weeks later. All radical prostatectomies were uncomplicated. The surgeons did not report any difficulties due to the previous laser ablation. All patient and procedure characteristics are shown in Table 2.

**Table 2:** Patient and procedure characteristics

Patient	Age (yr)	PSA (ng/mL)	Biopsy gleason score	Procedure time (min)	Ablation time (s)	Laser energy (*W*)	Final gleason score
1	66	9.3	3 + 4	84	91	12	3 + 2 (+4)
2	58	8.5	3 + 3	118	91	10	3 + 3
3	67	10.2	4 + 3	100	91	10	3 + 4
4	60	16.2	3 + 4	74	81	15	3 + 4 (+5)
5	70	5.7	3 + 2	59	73	11	3 + 2 (+5)

### Histopathological outcomes

**Fig. 3:**
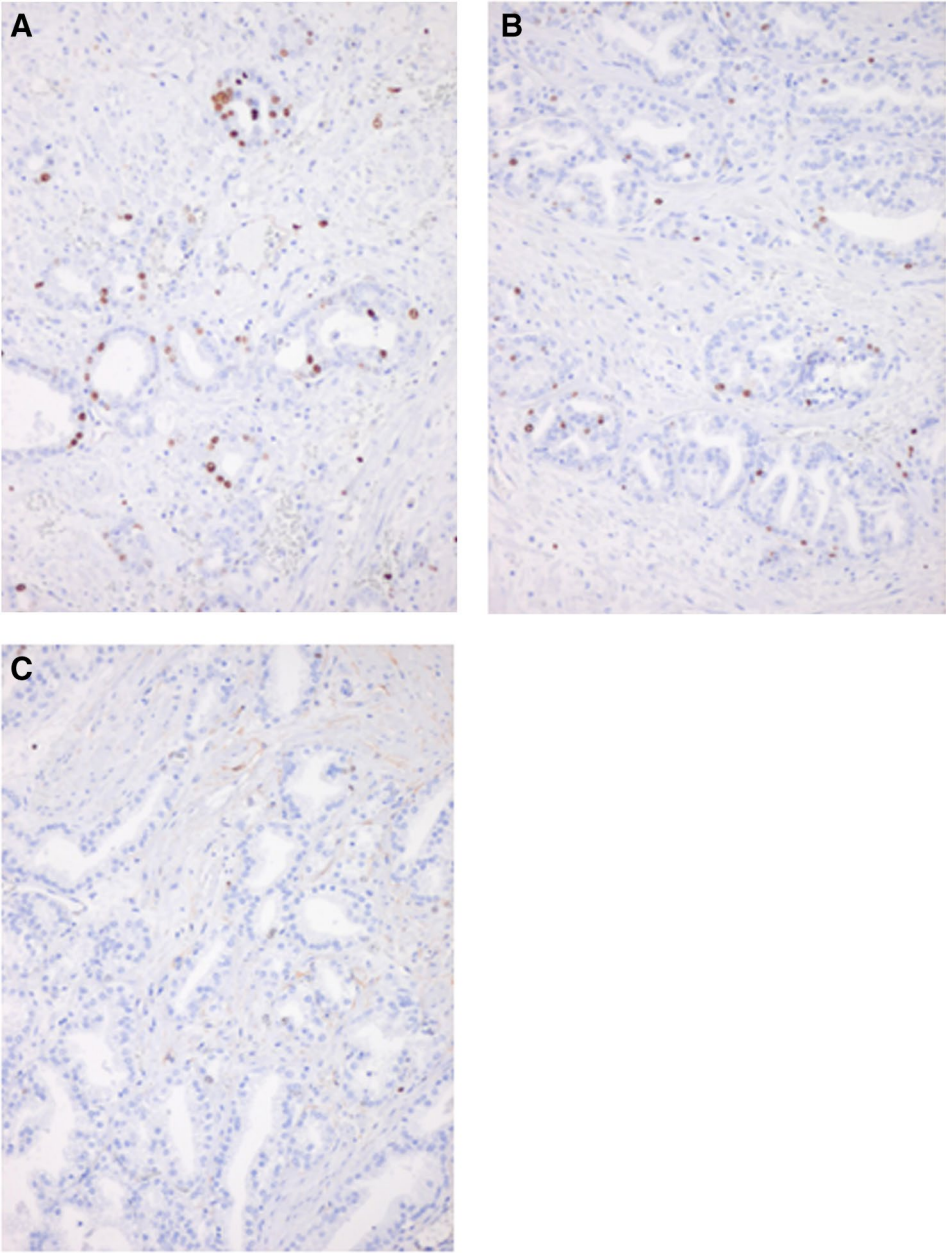
Histopathologic Ki67-stained slides in an axial plane through the prostate showing mitotic activity represented by the *brown* nuclei. **a** Perinecrotic area, showing increased mitotic activity. **b** Tumor invasion front, showing lower mitotic activity than in the perinecrotic area. **c** Center area of vital tumor, showing hardly any mitotic activity

The results of both pathologists were in concordance. A homogeneous necrotic area surrounded by a reactive perinecrotic zone of variable thickness (1–5 mm) was observed in all patients. The perinecrotic zone showed pre-existing prostatic tissue and tumor tissue with reactive changes, such as increased proliferation activity, neovascularisation with doubling of the microvessel density, and a variable lymphocytic infiltrate. Pre-existing prostate tubuli showed reactive epithelial changes, such as increased proliferation rate and squamous metaplasia. In the CK8/18, stain tumor tubuli were less resistant to the ablation than pre-existing tubuli, which extended further into the necrotic zone or perinecrotic zone. However, as indicated by the Ki67 stain, the tumor cells showed an increased mitotic index in the perinecrotic area (Fig. 3a), adjacent to the ablation area (Table 3). An increased mitotic activity was also seen at the tumor invasion front (Fig. 3b). The mitotic index was lowest in the center of the tumor (Fig. 3c).

**Table 3:** Mitotic index per area

Area	Mean MI/mm^2^ (range)
Paranecrotic tumor	99.3 (54–127)
Mid tumor	17.75 (1–58)
Opposite invasion front	41.3 (7–121)
Paranecrotic normal	85.0 (25–215)
Mid normal (BPH)	6.4 (1–21)

*MI* mitotic index, *BPH* benign prostate hyperplasia

### Volume comparison

All volumetric measurements per individual patient are shown in Table 4. The median ratio between the necrotic volume of the ablated tissue in the prostate specimen (Fig. 2d) and the measured ablation volume on the T1wCE MR images (Fig. 2c) was 0.80 (range, 0.40–2.09), and showed a significant volume correlation with a Pearson correlation coefficient of *r* = 0.94 (*p* = 0.018).

**Table 4:** Volumetric measurements per individual patient

Patient	Volume on damage-estimation map (cm^3^)	Volume on T1wCE images (cm^3^)	Volume on histopathology specimen corrected with shrinkage factor 1.15 (cm^3^)
1	3.30	1.00	1.06
2	1.93	0.22	0.00
3	3.65	0.17	0.43
4	2.32	1.10	1.67
5	1.46	0.12	0.06

The ablation volumes on the damage-estimation maps (Fig. 2b) derived from the MRI-temperature maps (Fig. 2a) were always larger than the ablation zones in the T1wCE MR images and the necrotic volumes in the histopathology specimen. The median ratios between those volumes were, respectively, 8.77 (range 2.11–21.47) and 5.85 (range 1.39–25.39), and were poorly correlated; *r* = 0.26 (*p* = 0.673) and *r* = 0.33 (*p* = 0.583).

## Discussion

The most important finding of our study was revealed by the histopathology results. All prostate specimens showed a homogeneous necrotic area surrounded by a perinecrotic area of 1–5 mm thickness. However, in this perinecrotic area, reactive changes were seen, as for example, neovascularization and an increased mitotic index. These processes were also seen in the tumor invasion front were the tumor invades in healthy tissue.

Stimulated growth of surviving tumor cells after incomplete thermal ablation (cryoablation or radiofrequency ablation) was observed earlier in renal tumor cells [22] and after incomplete radiofrequency ablation in liver cells [23]. Increased tumor cell proliferation was seen up to the end of the studies, respectively, 14 and 21 days after ablation, and is possibly caused by tissue damage, increased hypoxia, increased presence of heat-shock proteins, and inflammatory cells directly adjacent to the ablation area [22]. The clinical consequences of this effect remain unclear; in the worst-case scenario, it could facilitate tumor outgrowth; and in the best case scenario, the effect is only a temporary reaction on the cellular damage caused by the ablation. More research is needed to gather additional insight into these processes. Nevertheless, this highlights the importance of complete tumor ablation. To achieve this, the lesion size should be clearly visualized for optimal treatment planning and targeting and larger safety margins around the tumor should be employed. Le Nobin et al. [24] correlated visible prostate tumor volume on MRI with histology and concluded that a 9 mm non-capsular and a 3 mm capsular treatment margin should be applied to ensure complete tumor treatment.

In other ablate-resect studies, performed with MRI-guided FLA in canine prostates [25], in patients after ultrasound-guided FLA [12], or after HIFU [26, 27], increased tumor activity was not observed. A possible explanation for this difference might be that in all these studies, the total tumor was ablated and a less extensive histopathology work-up without Ki67 staining was performed.

The damage-estimation zones obviously overestimated the final necrotic volume in T1wCE images as well as the histopathology specimen, and showed poor volume correlation. The non-enhancing tissue on the post-ablation T1wCE MRI gave a better indication of the final histopathologic necrotic volume and showed a significant volume correlation. One of the reasons for the inconsistency between the ablation zones shown on the damage-estimation maps and the obtained ablation volumes in the histopathology specimen is probably due to tissue heterogeneity within the prostate. Furthermore, during laser ablation tissue properties, as for example, thermal conductivity, change due to temperature increase. Other factors which could have influenced the MRI-temperature mapping and, thus, the Arrhenius-based calculated volumes on the damage-estimation maps are the spatial resolution of the sequence, patient movement during scanning, and the inability of measuring temperature changes in extraprostatic fat tissue [28].

A study of Oto et al. [5] demonstrated successful MRI-guided FLA in nine patients. Urinary and sexual function did not significantly change afterwards. After 6 months, patients underwent MRI-guided biopsy of the ablation zone. In 78 %, no tumor was found, and in 22 %, a Gleason Score 6 was found. In another study by Lee et al. [16], 23 patients were treated. Thirteen of them underwent targeted biopsy of the ablation zone; only one (7.7 %) showed residual disease and was re-treated. No changes were seen in urinary or sexual functioning 6 months after the procedure. Recently, the results of 25 patients were reported by Lepor et al. [17]. Three months after treatment, the mean PSA decreased of 40 % and post-ablation MRI did not show signs of residual or recurrent PCa. Furthermore, targeted biopsy of the ablation zone was performed, and in 96 % of the ablation zones, no evidence of residual PCa was found.

This study had several limitations. First, a small sample size was used. Because of the large patient burden without any personal gain, the IRB committee considered it not ethical to treat more than five patients. Second, the volume calculations might have been influenced by several factors inherent to the difficulty of optimally matching MR images with pathology slices. Prostate specimens were sliced in a parallel plane used to perform the T1wCE sequence; however, there might have been small deviations. Furthermore, a tissue shrinkage factor of 1.15 was used for all the cases, because it was not possible to determine the shrinkage per individual prostate. Third, due to the small patient numbers and long inclusion period, a learning curve was present, especially influencing the procedure time.

Future steps will be to evaluate urinary and sexual function, quality-of-life, and oncological efficacy of MRI-guided FLA in patients with low-to-intermediate grade PCa on a longer term and large patient population.

In conclusion, this study demonstrated the importance of complete tumor ablation with a certain safety margin. Furthermore, T1wCE images are more reliable in determining the final ablation zone than damage-estimation maps derived from MRI-temperature maps. Although the initial results are promising, longer and thorough follow-up is needed to prove long-term outcome of this novel technique.

## References

[1] Siegel R, Ma J, Zou Z, Jemal A (2014). Cancer statistics, 2014. CA: Cancer J Clin.

[2] Sanda MG, Dunn RL, Michalski J (2008). Quality of life and satisfaction with outcome among prostate-cancer survivors. New Engl J Med.

[3] Resnick MJ, Koyama T, Fan KH (2013). Long-term functional outcomes after treatment for localized prostate cancer. New Engl J Med.

[4] Valerio M, Ahmed HU, Emberton M (2014). The role of focal therapy in the management of localised prostate cancer: a systematic review. Eur Urol.

[5] Oto A, Sethi I, Karczmar G (2013). MR imaging-guided focal laser ablation for prostate cancer: phase I trial. Radiology.

[6] Onik G, Vaughan D, Lotenfoe R, Dineen M, Brady J (2008). The “male lumpectomy”: focal therapy for prostate cancer using cryoablation results in 48 patients with at least 2-year follow-up. Urol Oncol.

[7] Bomers JG, Yakar D, Overduin CG (2013). MR imaging-guided focal cryoablation in patients with recurrent prostate cancer. Radiology.

[8] Barret E, Ahallal Y, Sanchez-Salas R (2013). Morbidity of focal therapy in the treatment of localized prostate cancer. Eur Urol.

[9] Napoli A, Anzidei M, De Nunzio C (2013). Real-time magnetic resonance-guided high-intensity focused ultrasound focal therapy for localised prostate cancer: preliminary experience. Eur Urol.

[10] Moore CM, Nathan TR, Lees WR (2006). Photodynamic therapy using meso tetra hydroxy phenyl chlorin (mTHPC) in early prostate cancer. Lasers Surg Med.

[11] Nguyen PL, Chen MH, Zhang Y (2012). Updated results of magnetic resonance imaging guided partial prostate brachytherapy for favorable risk prostate cancer: implications for focal therapy. J Urol.

[12] Lindner U, Lawrentschuk N, Weersink RA (2010). Focal laser ablation for prostate cancer followed by radical prostatectomy: validation of focal therapy and imaging accuracy. Eur Urol.

[13] Raz O, Haider MA, Davidson SR (2010). Real-time magnetic resonance imaging-guided focal laser therapy in patients with low-risk prostate cancer. Eur Urol.

[14] Woodrum DA, Gorny KR, Mynderse LA (2010). Feasibility of 3.0T magnetic resonance imaging-guided laser ablation of a cadaveric prostate. Urology.

[15] Woodrum DA, Mynderse LA, Gorny KR, Amrami KK, McNichols RJ, Callstrom MR (2011). 3.0T MR-guided laser ablation of a prostate cancer recurrence in the postsurgical prostate bed. J Vasc Interv Radiol: JVIR.

[16] Lee T, Mendhiratta N, Sperling D, Lepor H (2014). Focal laser ablation for localized prostate cancer: principles, clinical trials, and our initial experience. Rev Urol.

[17] Lepor H, Llukani E, Sperling D, Futterer JJ (2015). Complications, recovery, and early functional outcomes and oncologic control following in-bore focal laser ablation of prostate cancer. Eur Urol.

[18] Nash PA, Bruce JE, Indudhara R, Shinohara K (1996). Transrectal ultrasound guided prostatic nerve blockade eases systematic needle biopsy of the prostate. J Urol.

[19] Ishihara Y, Calderon A, Watanabe H (1995). A precise and fast temperature mapping using water proton chemical shift. Magn Reson Med.

[20] Sapareto SA, Dewey WC (1984). Thermal dose determination in cancer therapy. Int J Radiat Oncol Biol Phys.

[21] Jonmarker S, Valdman A, Lindberg A, Hellstrom M, Egevad L (2006). Tissue shrinkage after fixation with formalin injection of prostatectomy specimens. Virchows Archiv: An Int J Pathol.

[22] Kroeze SG, van Melick HH, Nijkamp MW (2012). Incomplete thermal ablation stimulates proliferation of residual renal carcinoma cells in a translational murine model. BJU Int.

[23] Nikfarjam M, Muralidharan V, Christophi C (2006). Altered growth patterns of colorectal liver metastases after thermal ablation. Surgery.

[24] Le Nobin J, Rosenkrantz AB, Villers A (2015). Image guided focal therapy for magnetic resonance imaging visible prostate cancer: defining a 3-dimensional treatment margin based on magnetic resonance imaging histology co-registration analysis. J Urol.

[25] Stafford RJ, Shetty A, Elliott AM (2010). Magnetic resonance guided, focal laser induced interstitial thermal therapy in a canine prostate model. JUrol.

[26] Chopra R, Colguhoun A, Burtnyk M (2012). MR imaging-controlled transurethral ultrasound therapy for conformal treatment of prostate tissue: initial feasibility in humans. Radiology.

[27] Beerlage HP, Van Leenders GJ, Oosterhof GO (1999). High-intensity focused ultrasound (HIFU) followed after one to two weeks by radical retropubic prostatectomy: results of a prospective study. Prostate.

[28] Rieke V, Butts Pauly K (2008). MR thermometry. J Magn Reson Imaging: JMRI.

